# Gender Discrimination of Flower Buds of Mature *Populus tomentosa* by HPLC Fingerprint Combined with Chemometrics

**DOI:** 10.1155/2022/1281521

**Published:** 2022-09-29

**Authors:** Zhuojun Li, Cui Wu, Bo Xu, Huijun Wang, Pingping Song, Zhenying Liu, Zhimao Chao

**Affiliations:** Institute of Chinese Materia Medica, China Academy of Chinese Medical Sciences, Beijing 100700, China

## Abstract

A high performance liquid chromatography-diode array detector (HPLC-DAD) was used to establish the HPLC fingerprint. Chemometrics methods were used to discriminate against the gender of flower buds of *Populus tomentosa* based on areas of common peaks calibrated with the HPLC fingerprint. The score plot of principal component analysis (PCA) showed a clear grouping trend (*R*^2^*X*, 0.753; *Q*^2^, 0.564) between female and male samples. Two groups were also well discriminated with orthogonal partial least squares-discriminant analysis (OPLS-DA) (*R*^2^*X*, 0.741; *R*^2^*Y*, 0.980; *Q*^2^, 0.970). As the hierarchical clustering analysis (HCA) heatmap showed, all samples were separated into two groups. Four compounds were screened out by S-plot and variable importance in projection (VIP > 1.0). Two of them were identified as siebolside B and tremulacin. This study demonstrated that HPLC fingerprints combined with chemometrics can be applied to discriminate against dioecious plants and screen differences, providing a reference for identifying the gender of dioecious plants.

## 1. Introduction

*Populus tomentosa* Carrière (Fam. Salicaceae) is a deciduous tree, which is widely planted in North China, such as Beijing. It is a common afforestation tree species, which can prevent the damage of wind and sand in the north. The leaves, barks, and male inflorescences can be used in traditional Chinese medicine (TCM). The dried male inflorescences of *P. tomentosa*, as the plant source of TCM Flos populi, are recorded in the People's Republic of China Pharmacopoeia and used to treat acute colitis and bacillary dysentery with the effect of reducing dampness and stopping dysentery [1]. The chemical constituents of *P. tomentosa* are mainly flavonoids, sterols, organic acids, phenols, and glycosides [2–4], which have anti-inflammatory, analgesic, antidiarrheal, antibacterial, and antioxidant effects [5–8]. The barks of mature *P. tomentosa* have the efficacy of clearing away heat and expelling dampness and are mainly used for the treatment of dysentery, leucorrhea, acute hepatitis, bronchitis, pneumonia, roundworms, and habitual constipation [9]. Flos populi is used as a veterinary medicine for the treatment of dysentery and diarrhea in cattle, sheep, and pigs [10–12]. In addition, fresh male inflorescences are used as food in China [13].

*P. tomentosa* is one of the dioecious plants that refers to seed plants with unisexual flowers and where the female and male flowers grow on different plants [14, 15]. In the last several years, many studies have focused mainly on physiological and biochemical indicators [16, 17] and molecular biotechnology [18, 19]. However, few studies have compared the differences between dioecious plants from the perspective of chemical constituents and their content [20]. There are some reports about the chemical constituents of *P. tomentosa*. The male barks of *P. tomentosa* have isolated and identified some compounds, such as siebolside B, sakuranetin, isograndidentatin A, and sakuranin [3]. The method of high-performance liquid chromatography (HPLC) fingerprint combined with chemometrics was used to study the differences in the chemical constituents and their content in the male and female barks of mature *P. tomentosa*, which showed that the content of four compounds was different, containing siebolside B, sakuranin, isograndidentatin A, and micranthoside. The content of micranthoside in the male samples was lower than that in the female samples. The content of sakuranin, siebolside B, and isograndidentatin A was higher in the male barks than in the female barks [21]. The differences in volatile components of mature *P. tomentosa* flower buds were studied by HS-SPME-GC-MS, which showed that the content of four compounds was different, containing benzyl benzoate, 2-cyclohexen-1-ketone, methyl benzoate, and benzoate. The content of benzyl benzoate, 2-cyclohexen-1-ketone, and methyl benzoate in the male samples was significantly lower than that in the female samples. The concentration of methyl benzoate in the male flower buds was remarkably lower than that in female flower buds [22].

The male inflorescences of *P. tomentosa* were used as TCM and food, so it is of great significance to discriminate against the gender of *P. tomentosa*. The flower buds are reproductive organs of *P. tomentosa*, and unlike with bark, the differences in chemical constituents should better reflect the correlation with their gender. The male and female inflorescences of *P. tomentosa* cannot be distinguished from the appearance of the trees except for the fruits and mature flowers. The relative expression of a full-length cDNA-denominated PtLFY in females was remarkably lower than that in males, which was found by using the RT-PCR technique [23]. However, there has been no report that shows differences in the nonvolatile components in the flower buds of *P. tomentosa* until now.

In this study, we collected 11 female and 11 male flower buds of mature *P. tomentosa* and established the HPLC fingerprint. Differential compounds between female and male flower buds were screened and identified with chemometrics and UPLC-Q-TOF/MS, respectively.

## 2. Materials and Methods

### 2.1. Apparatus and Reagents

An analytical Shimadzu LC-20AT apparatus (Shimadzu Corporation, Tokyo, Japan) was used for chromatographic fingerprints, equipped with a CBM-20A system controller, an LC-20AT pump, a CTO-10ASvp column oven, an SPD-M20A UV-vis detector, an SIL-20A autoinjector, a DGU-20A_5_ degasser, and a Shimadzu LC-solution work station. All MS data acquisitions were collected using a Vion IMS quadrupole time of flight (Q-TOF) configuration (Waters, Milford, MA, America) equipped with an electrospray ionization (ESI) source. An Ohaus CP224C electronic balance was manufactured by Ohaus Corporation (Tianjin, China). A DFT-50A portable high-speed disintegrator was purchased from Wenling Linda Machinery Corporation (Zhejiang, China). A DFD-700 double-row, four-hole electrothermal constant temperature water bath was purchased from Taisite Corporation (Tianjin, China). A D2012 table high-speed small centrifuge was produced by Scilogex Corporation (Rocky Hill, CT, America).

The HPLC grade methanol was purchased from Fisher Scientific (Fair Lawn, NJ, America). Purified water was purchased from Hangzhou Wahaha Group Co., Ltd. (Hangzhou, China). Analytical grade methanol was purchased from Fuyu Fine Chemicals Co., Ltd. (Tianjin, China). Our laboratory extracted, separated, and purified the reference standards of siebolside B, isograndidentatin A, and sakuranetin with more than 95% purity [3].

### 2.2. Sample Collection

The samples of 11 male flower buds (MFB) and 11 female flower buds (FFB) of mature *P. tomentosa* were collected on the 23rd–28th February 2020, in Beijing, China, which were located at 39°52′36″–39°57′17″ north latitude and 116°22′53″–116°26′57″ east longitude. The circumferences were from 85 to 197 cm at 1.0 m height. These flower buds were identified as the male and female flower buds of mature *P. tomentosa* (Fam. Salicaceae) by Prof. Zhimao Chao (Institute of Chinese Materia Medica, China Academy of Chinese Medical Sciences) according to the description in Flora of China (Editorial Board of Flora of China, 1984). All 22 voucher specimens (M1–M11 and F1–F11) were placed in the 1022 room of the Institute of Chinese Materia Medica, China Academy of Chinese Medical Sciences, Beijing, China.

### 2.3. Sample Preparation and Standard Solutions

All flower buds were dried in the shade, crushed into powder with a grinder, and passed through a 24-mesh sieve. The powder of 2.0 g was weighed precisely into a 50 mL conical flask, and then 25 mL of 70% methanol was added. They were weighed and refluxed at 80°C for 30 min and then were weighed again after cooling. The weight losses by refluxing were replaced with 70% methanol. Three standards were weighed appropriately and dissolved in 70% methanol. The sample and standard solutions were filtered through a 0.45 *μ*m membrane filter.

### 2.4. Chromatography Conditions

The HPLC chromatographic separation of the sample and standard solutions was performed on an Agilent 5 TC-C_18_ (2) column (4.6 × 250 mm, 5 *μ*m). The mobile phase consisted of methanol (A) and a 0.1% formic acid water solution (B). The HPLC elution condition was optimized as follows: 0–45 min: 10%–35%A; 45–110 min: 35%–58%A; 110–145 min: 58%–80%A; 145–150 min: 80%–100%A; and 150–160 min: 100%A. The column temperature was kept at 35°C. The flow rate was set at 1.0 mL/min. The detection wavelength was set at 287 nm. The injection volume was 10 *μ*L.

### 2.5. Mass Spectrometry Conditions

An Acquity UPLC BEH C_18_ column (2.1 mm × 100 mm, 1.7 *μ*m) was used in the ultra-performance liquid chromatography coupled with quadrupole time-of-flight mass spectrometry (UPLC-Q-TOF-MS). The mobile phase consisted of methanol (A) and 0.1% formic acid water (B). The UPLC elution condition was applied as follows: 0–5.6 min: 10%–35% A; 5.6–13.7 min: 35%–58% A; 13.7–18 min: 58%–80% A; 18–21 min: 80%–100% A; 21–25 min: 10% A. The column temperature was kept at 35°C. The flow rate of the mobile phase was 0.3 mL/min. The detection wavelength was set at 287 nm. The injection volume was 2 *μ*L. UPLC-Q-TOF-MS was operated in the positive and negative modes with a scanning range of *m/z* 50–1200. The capillary voltage and cone voltage were 3.0 kV and 30.0 kV, respectively. The ion source temperature was set at 120°C. The ion desolvation temperature was set at 450°C. The scan time of the data collection was 0.2 s. The flow rates of cone gas and desolvation gas (N_2_) were set at 50 L/h and 1000 L/h, respectively.

### 2.6. Validation of the HPLC Method

All sample solutions were prepared according to 2.3 and analyzed in the chromatography system. The precision of the chromatographic method was determined by analyzing the same sample solution injected six times in a single day. The stability was determined by analyzing the same sample solution injected after 0, 3, 6, 9, 16, and 24 hours. The repeatability was determined by analyzing six replicates prepared from the same sample. The relative standard deviation (RSD, %) values of the relative retention time (RRT) and the relative peak area (RPA) of each common peak were calculated to estimate precision, stability, and repeatability.

### 2.7. Statistical Analysis

The raw chromatographic data files of 22 flower buds were transformed into AIA format files. Then, these files were imported into the software “Similarity Evaluation System for Chromatographic Fingerprint of TCM” (Version 2004A, Committee for the Pharmacopoeia of PR China). One of the samples was randomly chosen as the reference chromatogram. The control fingerprint, which can represent the characteristics of samples, was generated by using an average method and performing multipoint correction and automatic match. The similarities of the HPLC fingerprint of the samples were obtained, which were based on the reference fingerprint. The peak areas of all common peaks were utilized to perform the chemometrics.

Chemometrics employs unsupervised and supervised models. Principal component analysis (PCA) and orthogonal partial least squares discriminant analysis (OPLS-DA) were performed using the software SIMCA-P version 14.1 (Umetrics) to discriminate between MFB and FFB of *P. tomentosa*. The screened compounds were analyzed by a hierarchical clustering analysis (HCA) heatmap performed by MetaboAnalyst (https://www.metaboanalyst.ca/MetaboAnalyst/home.xhtml).

Firstly, unsupervised PCA was used to analyze the clustering of samples and yield a score plot. Subsequently, supervised OPLS-DA was used to estimate sample grouping and obtain the differential compounds between male and female samples. Then, the permutation test was to prevent the OPLS-DA model from overfitting. The differential variables were selected by S-Plot and variable importance for the projection (VIP) values (VIP > 1) [24–26]. The student's *t*-test was carried out by the SPSS 25.0 software, which could verify that the differential variables were significantly different. Additionally, an HCA heatmap was applied to observe the clustering of samples, which illustrated the content of differential compounds in different groups of samples.

To identify the differential compounds, sample F4 was analyzed by the UPLC-Q-TOF/MS (Waters, Milford, MA, America). 292 compounds published in the literature about *P. tomentosa* and other species of the *Populus* genus were downloaded as structure files (mol and .sdf) from Chemspider (https://www.chemspider.com) and PubChem (https://pubchem.ncbi.nlm.nih.gov/). All files were integrated into a database by Progenesis SDF Studio. MS data were imported into Progenesis QI and matched with the database. Each mass spectrum was manually analyzed with Waters MassLynx V4.1 software to verify whether the compounds predicted by the software were correct.

## 3. Results and Discussion

### 3.1. Optimization of Sample Preparation

In this study, the extraction solvents (methanol and a serial concentration of methanol/water) and the sample-to-solvent ratios (1 : 10, 1 : 12.5, 1 : 20, and 1 : 30 g/mL) were investigated to extract the sample. The results suggested that 70% methanol and a ratio of 1 : 12.5 g/mL were better than other extraction solvents according to the numbers of chromatographic peaks and peak areas. Therefore, they were selected for further experiments. Ultrasonic and reflux extractions were studied to obtain the best extraction efficiency. The results suggested that the reflux extraction was better than the ultrasonic extraction. Extraction time (30 min, 60 min, 90 min, and 120 min) was also tested and evaluated. The numbers of chromatographic peaks, peak areas, and extraction efficiency were comprehensively considered for evaluation (Figure S1). Finally, 2.0 g of the sample was selected and 25 mL of 70% methanol was added for refluxing for 30 min to prepare the sample solutions.

### 3.2. Optimization of HPLC Conditions

The HPLC mobile phases (methanol-water and methanol-0.1% formic acid water solution) and some analytical columns (Diamonsil C_18_ (1), Diamonsil C_18_ (2), Diamonsil Plus C_18_, Agilent5 TC-C_18_ (2), and Agilent ZORBAX SB-C_18_) were also optimized. The baseline, chromatographic peak shape, and analysis time were comprehensively considered for evaluation (Figure S2). Finally, methanol/water of mobile phase with a flow rate of 1.0 mL/min, Agilent5 TC-C_18_ (2) (4.6 mm × 250 mm, 5 *μ*m), column temperature at 35°C, and injection volume of 10 *μ*L were selected.

### 3.3. Evaluation of Validation of the HPLC Method

The precision, stability, and repeatability were determined using the method of HPLC-DAD and assessed by the RSDs of RRT and RPA of the common peaks. All of the RSD results were below 3.0% (Table 1), indicating that the HPLC method was a stable and feasible method for fingerprint analyses.

### 3.4. HPLC Fingerprint

**Figure 1:**
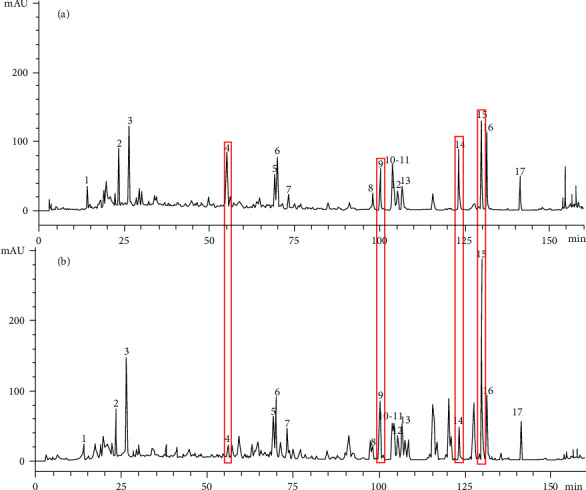
The example of HPLC chromatograms of M2 (a) and F4 (b). The chromatographic peaks of the known compounds: 4, siebolside B; 5, isograndidentatin A; and 12, sakuranetin. The *X* axis refers to the retention time (t/min). The *Y* axis refers to the response value (mAU).

**Figure 2:**
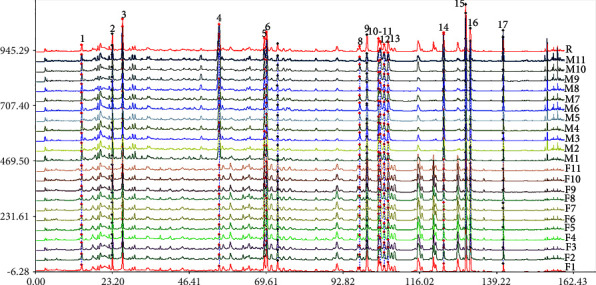
HPLC fingerprints of MFB and FFB of *P. tomentosa*. F1–F11 are female flower bud samples. M1–M11 are male flower bud samples. *R* refers to the control fingerprint. The *X* axis refers to the retention time (t/min). The *Y* axis refers to the response value (mAU). The peaks 1–17 are the main common peaks.

The chromatograms of 22 flower buds of *P. tomentosa* were aligned and matched to obtain the HPLC fingerprints by the Similarity Evaluation System for Chromatographic Fingerprint of TCM. The examples of HPLC chromatograms of F4 and M2 are shown in Figure 1. The HPLC fingerprints of all samples and the control fingerprint are shown in Figure 2. Seventeen common peaks were calibrated. Peak 4 was determined as the reference fingerprint peak that was used to calculate RRT and RPA. Seventeen peaks were determined as the common peaks in the fingerprints of male and female flower buds of *P. tomentosa*. The peaks 4, 5, and 12 were identified as siebolside B, isograndidentatin A, and sakuranetin, respectively, based on the standards. The similarity of 11 female and 11 male samples was 0.978–0.999 and 0.943–0.985 (Tables S1 and S2), respectively, which indicated that the HPLC fingerprint of MFB and FFB of *P. tomentosa* was highly similar (similarity > 0.9) [27]. The similarities of 22 samples are displayed in Table S3, which indicates that the male and female samples were difficult to classify by HPLC fingerprint.

### 3.5. PCA and OPLS-DA

**Figure 3:**
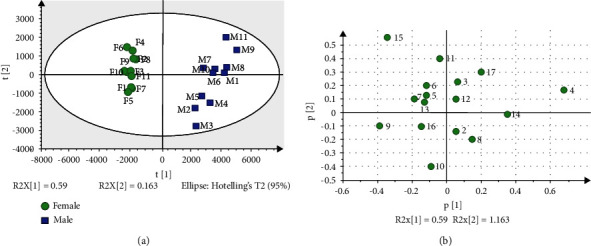
PCA score plot and loading scatter plot based on the peak areas of 17 common peaks of MFB and FFB of *P. tomentosa*. (a) Score plot of the PCA model. Boxes refer to MFB of *P. tomentosa*, and circles refer to FFB of *P. tomentosa*. The *X* axis refers to the scores of the first principal component (PC1). The *Y* axis refers to the scores of the second principal component (PC2). The variances accounted by PC1 and PC2 were 59.0% and 16.3%, respectively. (b) Loading scatter plot of the PCA model.

To analyze the difference between FFB and MFB of *P. tomentosa*, the areas of 17 common peaks of 22 flower buds were imported into SIMCA-P version 14.1 software to perform unsupervised PCA analysis. The score plot and loading scatter plot of PCA are shown in Figure 3. The first and second PCs reflected 59.0% and 16.3% of the sample information, respectively. The first two PCs accounted for 75.3% (*R*^2^*X*) of the total variance and could be applied to classify the samples, which replaced the 17 original compounds and reflected the compound information of the samples. The *Q*^2^ was 0.564, which indicated that the PCA model had good predictive ability (*Q*^2^ ≥ 0.50) [28]. As shown in Figure 3(a), FFB (on the left) and MFB (on the right) were clustered into one group, respectively. The distribution of 17 peaks was relatively scattered ( Figure 3(b)). Peaks 4, 9, 14, and 15 provided the main difference between female samples and male samples. Therefore, these results demonstrated that there were differences between MFB and FFB of *P. tomentosa* in the chemical constituents.

**Figure 4:**
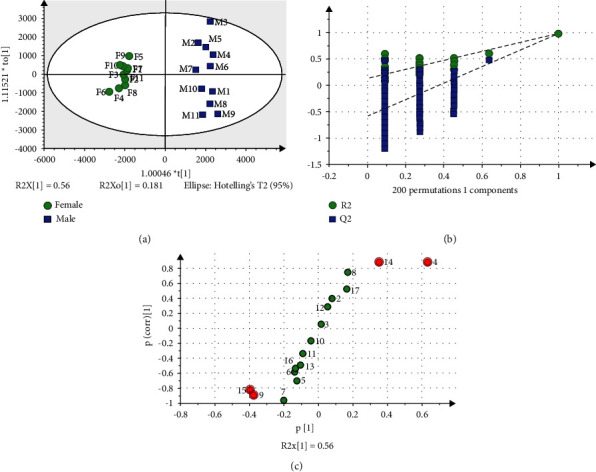
OPLS-DA model for the classification of MFB and FFB of *P. tomentosa*. (a) Score plot of the OPLS-DA model. Boxes refer to MFB of *P. tomentosa*, and circles refer to FFB of *P. tomentosa*. The *X* axis refers to the scores of PCs. The *Y* axis refers to the scores of orthogonal components. (b) 200 permutation tests of the OPLS-DA model. The *X* axis represents the correlation between the permuted model and the original model. The *Y* axis represents *R*^2^ and *Q*^2^ of the permuted models. (c) *S*-plot of the OPLS-DA model. The *X* axis represents the correlation coefficient between PCs and common compounds. The *Y* axis represents VIP values.

To screen out the differential variables between male and female samples, the areas of 17 common peaks were imported into SIMCA-P version 14.1 software to perform supervised OPLS-DA analysis. The score plot of OPLS-DA is shown in Figure 4(a). The result of OPLS-DA showed that the *R*^2^*X* was 0.741, which demonstrated that 74.1% of the variance could be modeled by these chosen compounds on the *X* axis. The *R*^2^*Y* and *Q*^2^ are 0.980 and 0.970, respectively. They were close to 1, indicating that the OPLS-DA model was fully fitted and had great predictivity [29–31]. The permutation test was performed based on the OPLS-DA model to further test whether the OPLS-DA model was reliable. Two‐hundred permutation test results showed that the intercepts of *R*^2^ and *Q*^2^ were 0.134 and −0.582 on the *Y* axis. As shown in Figure 4(b), the *R*^2^ and *Q*^2^ on the left, which were generated by random permutations, were less than the original values on the right. The intercept of the regression line of *Q*^2^ on the *Y* axis was below zero. Therefore, this model had no overfitting and was reliable [32, 33]. The *S*-plot is shown in Figure 4(c). Based on the *S*-plot (|*p*[1]| ≥ 0.2 and |*p*(corr)[1]| ≥ 0.8) and the VIP values (VIP > 1), four peaks of 4, 9, 14, and 15 were screened out. The results of the student's *t*-test showed that there was a significant difference (*p* < 0.05) in these four peaks between FFB and MFB of *P. tomentosa*.

### 3.6. HCA Heatmap

**Figure 5:**
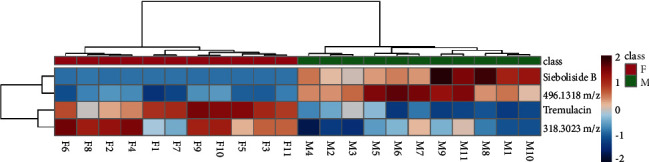
HCA heatmap analyses of 4 peak content in MFB and FFB of *P. tomentosa*. The *X* axis refers to *P. tomentosa* samples. The *Y* axis refers to the screened compounds. The red and blue colors in the plot refer to higher and lower compound content than the mean value, respectively.

Four screened compounds were plotted on the HCA heatmap (Figure 5) to get an intuitive overview of the differential compounds between the male and female samples. The samples were classified into two groups: the content of siebolside B and peak 14 (496.1318 *m/z*) were higher in the male samples, and the content of tremulacin and peak 15 (318.3023 *m/z*) were higher in the female samples.

According to the standard and literature, combined with the polarity, 14 compounds were identified in Table 2 and Table S4 (Figures S3–S18) [21, 34–40]. The retention times at 8.91, 13.76, 17.37, and 18.14 min corresponded to the peaks 4, 9, 14, and 15 in the HPLC fingerprint by comparing the peak area, polarity, and spectrum. As shown in Table 2, two of the four screened compounds were identified, containing siebolside B and tremulacin. These two components might be gender markers of *P. tomentosa*. This provided the reference for further research and utilization of *P. tomentosa*. The pharmacological activity of siebolside B is allergy-preventive [41]. Tremulacin has anti-inflammatory, antiviral, and antioxidant activities and can inhibit xanthine oxidase activity [41–44]. As shown in Figure 5, the content of siebolside B in MFB was higher than that in FFB. The content of siebolside B in the male barks of *P. tomentosa* was also higher than that in the female barks of *P. tomentosa*. The male inflorescences of *P. tomentosa* are the plant source of Flos populi [21]. Therefore, siebolside B may be the important bioactive compound in male *P. tomentosa*. There have been a few studies on siebolside B so far. This has guiding significance for the following research.

Fourteen compounds were identified in F4 by UPLC-Q-TOF/MS. Catechin, sakuranetin, and isosakuranetin belong to flavonoids. Flavonoids have antioxidant, anti-inflammatory, analgesic, anticancer, and other activities [45], which are widely found in medicinal plants. Siebolside B, tremulacin, salicin, salicortin, tremuloidin, isograndidentatin A, and benzyl caffeate belong to phenolic glycosides. Phenols have antioxidant, anti-inflammatory, antibacterial, and antifungal activities [46, 47]. The main active compounds of Flos populi are flavonoids and phenols. This can provide a reference for the expansion of the use of *P. tomentosa* flowers.

The method of HPLC fingerprint combined with chemometrics is simple, fast, and accurate. It accurately distinguished the gender of flower buds of *P. tomentosa* in this study and enriched research on dioecious plants. In late spring and early summer, a large number of poplar catkins of *P. tomentosa* are scattered in the air, causing people to have symptoms such as cough and allergies, which causes great trouble in people's lives [48, 49]. This method can be applied to identify the gender before the flower buds of *P. tomentosa* mature. Poplar catkins can be treated by some physical measures, such as artificially trimming female flower buds. This will provide a reference for reducing poplar catkin pollution.

## 4. Conclusions

In this study, gender discrimination of flower buds of mature *P. tomentosa* was achieved by using an HPLC fingerprint combined with chemometrics. The female and male flower buds of *P. tomentosa* were clearly discriminated by PCA, OPLS-DA, and HCA heatmap. Furthermore, 15 compounds were identified, and 4 compounds were successfully screened out by S-plot and VIP values, whose content was significantly different in the female and male samples. Therefore, this study provided a reference for the gender identification of dioecious plants and enriched research on dioecious plants.

## Figures and Tables

**Table 1:** RSD results of precision, stability, and repeatability.

Peak	Precision (*n* = 6)		Stability (*n* = 6)		Repeatability (*n* = 6)	
	RSD of RRT (%)	RSD of RPA (%)	RSD of RRT (%)	RSD of RPA (%)	RSD of RRT (%)	RSD of RPA (%)
1	0.19	1.28	0.17	1.80	0.18	2.08
2	0.22	1.88	0.16	2.73	0.38	2.03
3	0.16	2.36	0.10	2.03	0.26	1.72
4	0.00	0.00	0.00	0.00	0.00	0.00
5	0.04	1.41	0.03	1.39	0.05	1.76
6	0.04	1.61	0.03	1.84	0.06	2.04
7	0.06	2.36	0.05	2.69	0.04	2.46
8	0.09	2.15	0.06	2.28	0.11	1.97
9	0.08	1.24	0.06	1.63	0.12	1.32
10	0.11	2.88	0.10	2.75	0.10	2.99
11	0.10	1.15	0.06	2.40	0.13	1.76
12	0.12	2.94	0.11	2.78	0.08	2.05
13	0.09	1.70	0.05	1.67	0.14	1.87
14	0.11	1.65	0.09	1.52	0.19	2.46
15	0.13	1.01	0.13	1.07	0.21	1.56
16	0.12	1.27	0.13	1.32	0.19	0.76
17	0.16	1.47	0.18	1.47	0.24	1.76

RSD of RRT refers to the relative standard deviation values of the relative retention time; RSD of RPA refers to the relative standard deviation values of the relative peak areas.

**Table 2:** Four screen compounds identified by UPLC-Q-TOF/MS.

Peak	Rt (min)	Assignment	Formula	ESI (+)		ESI (−)		Reference
				MS^1^ (ppm)	MS^2^	MS^1^ (ppm)	MS^2^	
4	8.91	Siebolside B^a^	C_20_H_22_O_9_	429.1171 [M + Na]^+^ (2.1); 407.1346 [M + H]^+^ (1.0)	245.0844; 123.0473	405.1192 [M − H]^−^ (1.5)	243.0616; 121.0288	Standard

9	13.76	Tremulacin^b^	C_27_H_28_O_11_	551.1535 [M + Na]^+^ (1.1); 567.1257 [M + K]^+^ (−2.1); 529.1698 1697 [M + H]^+^ (−2.5)	289.0713; 160.9955; 179.0350	527.1561 [M − H]^−^ (1.5)	405.1185; 155.0341; 137.0236; 121.0288	[34]

14	17.37	NI	C_31_H_23_NO_3_	496.1318 [M + K]^+^ (0.6)	301.0737; 213.0983; 147.0482	—	—	—

15	18.14	NI	C_18_H_40_NO_3_	318.3023 [M + H]^+^ (2.5)	256.2657; 186.2247	—	—	—

a: identified by reference standard. b: identified by literature. NI: not identified.

## Data Availability

The data used to support the findings of this study are included within the article.

## Supplementary Materials

Supplementary Materials Table S1: similarity evaluation of 11 male flower buds (MFB) of *P. tomentosa*. Table S2: similarity evaluation of 11 female flower buds (FFB) of *P. tomentosa*. Table S3: similarity evaluation of 22 flower buds of *P. tomentosa*. Table S4: other compounds of the sample F4 identified by UPLC-Q-TOF/MS. Figure S1: optimization of the sample preparation: (A) optimization of the extraction solvents; (B) optimization of the sample-to-solvent ratios; (C) optimization of the extraction methods; the picture above depicts reflux extraction, while the picture below depicts ultrasonic extraction; and (D) optimization of the extraction time. Figure S2: optimization of HPLC Conditions: (A) optimization of the mobile phases: the picture on the left depicts the methanol-water of the mobile phase, and the picture on the right depicts the methanol-0.1% formic acid in the water of the mobile phase; (B) optimization of the analytical columns: the B1 is about the column of Diamonsil C_18_ (1), the B2 is about the column of Diamonsil C_18_ (2), the B3 is about the column of Diamonsil Plus C_18_, the B4 is about the column of Agilent 5 TC-C_18_ (2), and the B5 is about the column of Agilent ZORBAX SB-C_18_. Figure S3: the MS^1^ and MS^2^ spectrograms of the sample in the positive and negative mode: (A) the MS^1^ spectrogram at 3.56 min in the positive mode; (B) the MS^2^ spectrogram at 3.56 min in the positive mode; the source ion of MS^2^ is 309.0961 *m/z*; (C) the MS^1^ spectrogram in the negative mode; and (D) the MS^2^ spectrogram in the negative mode. Figure S4: the MS^1^ and MS^2^ spectrograms of the sample in the positive and negative mode: (A) the MS^1^ spectrogram at 4.01 min in the positive mode; (B) the MS^2^ spectrogram at 4.01 min in the positive mode; the source ion of MS^2^ is 291.0891 *m/z*; (C) the MS^1^ spectrogram in the negative mode; and (D) the MS^2^ spectrogram in the negative mode. Figure S5: the MS^1^ and MS^2^ spectrograms of the sample in the positive and negative mode: (A) the MS^1^ spectrogram at 7.55 min in the positive mode; (B) the MS^2^ spectrogram at 7.55 min in the positive mode; the source ion of MS^2^ is 425.1415 *m/z*; (C) the MS^1^ spectrogram in the negative mode; and (D) the MS^2^ spectrogram in the negative mode. Figure S6: the MS^1^ and MS^2^ spectrograms of siebolside B and sample in the positive and negative mode: (A) the MS^1^ spectrogram of siebolside B in the positive mode; (B) the MS^2^ spectrogram of siebolside B in the positive mode; the source ion of MS^2^ is 407.1359 *m/z*; (C) the MS^1^ spectrogram of peak No. 4 at 8.91 min in the sample in the positive mode; (D) the MS^2^ spectrogram of peak No. 4 at 8.91 min in the sample in the positive mode; the source ion of MS^2^ is 407.1313 *m/z*; (E) the MS^1^ spectrogram in the sample in the negative mode; (F) the MS^2^ spectrogram in the sample in the negative mode; and (G) the structure of siebolside B. Figure S7: the MS and MS/MS spectrograms of the sample in the positive and negative mode: (A) the MS spectrogram at 10.47 min in the positive mode; (B) the MS/MS spectrogram at 10.47 min in the positive mode; the source ion of MS^2^ is 425.1798 *m/z*; (C) the MS^1^ spectrogram in the negative mode; and (D) the MS^2^ spectrogram in the negative mode. Figure S8: the MS^1^ and MS^2^ spectrograms of the sample in the positive and negative mode: (A) the MS^1^ spectrogram at 10.65 min in the positive mode; (B) the MS^2^ spectrogram at 10.65 min in the positive mode; the source ion of MS^2^ is 425.1831 *m/z*; (C) the MS^1^ spectrogram in the negative mode; and (D) the MS^2^ spectrogram in the negative mode. Figure S9: the MS^1^ and MS^2^ spectrograms of the sample in the positive and negative mode: (A) the MS^1^ spectrogram at 10.92 min in the positive mode; (B) the MS^2^ spectrogram at 10.92 min in the positive mode; the source ion of MS^2^ is 425.1808 *m/z*; (C) the MS^1^ spectrogram in the negative mode; and (D) the MS^2^ spectrogram in the negative mode. Figure S10: the MS^1^ and MS^2^ spectrograms of the sample in the positive and negative mode: (A) the MS^1^ spectrogram at 11.21 min in the positive mode; (B) the MS^2^ spectrogram at 11.21 min in the positive mode; the source ion of MS^2^ is 413.1221 *m/z*; (C) the MS^1^ spectrogram in the negative mode; and (D) the MS^2^ spectrogram in the negative mode. Figure S11: the MS^1^ and MS^2^ spectrograms of the sample in the positive and negative mode: (A) the MS^1^ spectrogram of peak 9 at 13.76 min in the positive mode; (B) the MS^2^ spectrogram of peak 9 at 13.76 min in the positive mode; the source ion of MS^2^ is 551.1531 *m/z*; (C) the MS^1^ spectrogram in the negative mode; (D) the MS^2^ spectrogram in the negative mode; and (E) the structure of peak 9. Figure S12: the MS^1^ and MS^2^ spectrograms of the sample in the positive and negative mode: (A) the MS^1^ spectrogram at 16.01 min in the positive mode; (B) the MS^2^ spectrogram at 16.01 min in the positive mode; the source ion of MS^2^ is 287.0927 *m/z*; (C) the MS^1^ spectrogram in the negative mode; and (D) the MS^2^ spectrogram in the negative mode. Figure S13: the MS^1^ and MS^2^ spectrograms of the sample in the positive and negative mode: (A) Tthe MS^1^ spectrogram at 16.07 min in the positive mode; (B) the MS^2^ spectrogram at 16.07 min in the positive mode; the source ion of MS^2^ is 287.0934 *m/z*; (C) the MS^1^ spectrogram in the negative mode; and (D) the MS^2^ spectrogram in the negative mode. Figure S14: the MS^1^ and MS^2^ spectrograms of the sample in the positive mode: (A) the MS^1^ spectrogram of peak 14 at 17.37 min; and (B) the MS^2^ spectrogram of peak 14 at 17.37 min the source ion of MS^2^ is 496.1318 *m/z*. Figure S15: the MS^1^ and MS^2^ spectrograms of the sample in the positive mode: (A) the MS^1^ spectrogram of peak 15 at 18.14 min; and (B) the MS^2^ spectrogram of peak 15 at 18.14 min; the source ion of MS^2^ is 318.3023 *m/z*. Figure S16: the MS^1^ and MS^2^ spectrograms of the sample in the positive mode: (A) the MS^1^ spectrograms at 18.71 min; and (B) the MS^2^ spectrogram at 18.71 min. Figure S17: the MS^1^ and MS^2^ spectrograms of the sample in the positive mode: (A) the MS^1^ spectrograms at 19.81 min; and (B) the MS^2^ spectrogram at 19.81 min. Figure S18: the MS^1^ and MS^2^ spectrograms of the sample in the positive mode: (A) the MS^1^ spectrograms at 20.00 min; and (B) the MS^2^ spectrogram at 20.00 min.

## References

[1] National Pharmacopoeia Committee (1978). *Pharmacopoeia of the People’s Republic of China*.

[2] Lin M., Li S. Z. (1993). Study on chemical constituents of *Populus tomentosa*. *Acta Pharmaceutica Sinica*.

[3] Liu H., Chao Z., Wu X., Tan Z., Wang C., Sun W. (2012). Study on chemical constituents of *Populus tomentosa*. *China Journal of Chinese Materia Medica*.

[4] Hou Y., Zhang G., Cui H. (2017). Chemical constituents from the male anthotaxy of *Populus tomentosa* Carr. *Journal of International Pharmaceutical Research*.

[5] Xu Q., Shen Z., Wang Y. (2013). Anti-diarrhoeal and anti-microbial activity of *Flos populi* (male inflorescence of *Populus tomentosa* Carriere) aqueous extracts. *Journal of Ethnopharmacology*.

[6] Xu Q., Wang Y., Guo S., Shen Z., Wang Y., Yang L. (2014). Anti-inflammatory and analgesic activity of aqueous extract of *Flos populi*. *Journal of Ethnopharmacology*.

[7] Zhao Y., Tang G., Cai E., Liu S., Zhang L., Wang S. (2014). Hypolipidaemic and antioxidant properties of ethanol extract from *Flos populi*. *Natural Product Research*.

[8] Ni H., Muhammad I., Li J., Wang B., Sheng Z. (2019). *In vitro* and *in vivo* antioxidant activities of the flavonoid-rich extract from *Flos populus*. *Pakistan journal of pharmaceutical sciences*.

[9] Nanjing University of Chinese Medicine (2006). *Dictionary of Traditional Chinese Medicine*.

[10] Ma J. (2014). Effect of *Flos populi* in treatment of lamb dysentery. *Today Animal Husbandry Veterinary Medicine*.

[11] Song W., Guo J. (2016). 936 cases of calf diarrhea treated by self-made dysentery trigeminal. *Contemporary Animal Husbandry*.

[12] Zheng Q. (2020). Experience in prevention and treatment of piglet diarrhea. *Zhejiang Journal Animal Science and Veterinary Medicine*.

[13] Li S., Wang X., Ma G., Shi X., Deng Y., Geng Y. (2004). Analysis of nutritional composition of Chinese white poplar male anthotaxy. *Shandong Science*.

[14] Liu M., Korpelainen H., Li C. Y. (2021). Sexual differences and sex ratios of dioecious plants under stressful environments. *Journal of Plant Ecology*.

[15] Ye X., Zhao X., Sun Y. (2021). The underlying molecular conservation and diversification of dioecious flower and leaf buds provide insights into the development, dormancy breaking, flowering, and sex association of willows. *Plant Physiology and Biochemistry*.

[16] Feng S., Sun H., Ma H. (2020). Sexual differences in physiological and transcriptional responses to salinity stress of *Salix linearistipularis*. *Frontiers of Plant Science*.

[17] Chen J., Liu Q., Yu L., Korpelainen H., Niinemets U., Li C. Y. (2021). Elevated temperature and CO_2_ interactively modulate sexual competition and ecophysiological responses of dioecious *Populus cathayana*. *Forest Ecology and Management*.

[18] Neves C. J., Matzrafi M., Thiele M., Lorant A., Mesgaran M. B., Stetter M. G. (2020). Male linked genomic region determines sex in dioecious amaranthus palmeri. *Journal of Heredity*.

[19] Melnikova N. V., Pushkova E. N., Dvorianinova E. M. (2021). Genome assembly and sex-determining region of male and female *Populus* x *sibirica*. *Frontiers of Plant Science*.

[20] Liu H., Chao Z., Tan Z., Wu X. (2012). Material basis analysis and research technology of dioecious medicinal plants. *Chinese Journal of Information on Traditional Chinese Medicine*.

[21] Wu C., Xu B., Li Z., Song P., Chao Z. (2021). Gender discrimination of *Populus tomentosa* barks by HPLC fingerprint combined with multivariate statistics. *Plant Direct*.

[22] Xu L., Liu H., Ma Y., Wu C., Li R., Chao Z. (2019). Comparative study of volatile components from male and female flower buds of *Populus* x *tomentosa* by HS-SPME-GC-MS. *Natural Product Research*.

[23] An X., Wang D., Wang Z. (2010). Expression profile of PtLFY in floral bud development associated with floral bud morphological differentiation in *Populus tomentosa*. *Scientia Silvae Sinicae*.

[24] Wang H., Cao X., Yuan Z., Guo G. (2021). Untargeted metabolomics coupled with chemometrics approach for Xinyang Maojian green tea with cultivar, elevation and processing variations. *Food Chemistry*.

[25] Zhou B. X., Ma B. S., Ma C. Q. (2022). Classification of Pu-erh ripened teas and their differences in chemical constituents and antioxidant capacity. *LWT-Food Science and Technology*.

[26] Huang Y., Cheng X., Wu W. (2020). Evaluation of different processed fructus gardeniae based on fingerprint and multivariate statistical analysis. *Journal of Guangdong Pharmaceutical University*.

[27] Xu S. Z., Yang G. J., Feng F. (2017). Investigation of distinction chemical markers for rhubarb authentication based on high-performance liquid chromatography-time-of-flight mass spectrometry and multivariate statistical analysis. *Food Analytical Methods*.

[28] Mao Q., Bai M., Xu J. D. (2014). Discrimination of leaves of *Panax ginseng* and *P. quinquefolius* by ultra high performance liquid chromatography quadrupole/time-of-flight mass spectrometry based metabolomics approach. *Journal of Pharmaceutical and Biomedical Analysis*.

[29] Liang H. Z., Du Z. Y., Yuan S. (2021). Comparison of *Murraya exotica* and *Murraya paniculata* by fingerprint analysis coupled with chemometrics and network pharmacology methods. *Chinese Journal of Natural Medicines*.

[30] Yang M., Zhao Y., Qin Y., Xu R., Yang Z., Peng H. (2021). Untargeted metabolomics and targeted quantitative analysis of temporal and spatial variations in specialized metabolites accumulation in *Poria cocos* (Schw.) wolf (Fushen). *Frontiers of Plant Science*.

[31] Zhao Y., Zhao Y. Y., Du Y., Kang J. S. (2019). Characterization and classification of three common Bambusoideae species in Korea by an HPLC-based analytical platform coupled with multivariate statistical analysis. *Industrial Crops and Products*.

[32] Li R., Li J., Xu C. (2021). Identification of atractylodes macrocephala Koidz. from different areas by GC-MS fingerprint and multivariate statistical analysis. *Journal of Guangdong Pharmaceutical University*.

[33] Bao X., Jiang X., Ma J., Wang X., Zhou Q. (2020). Bioavailability and pharmacokinetics of anisatin in mouse blood by ultra-performance liquid chromatography-tandem mass spectrometry. *BioMed Research International*.

[34] Medana C., Carbone F., Aigotti R., Appendino G., Baiocchi C. (2008). Selective analysis of phenolic compounds in propolis by HPLC-MS/MS. *Phytochemical Analysis*.

[35] Carvalho D., Curto A., Guido L. (2015). Determination of phenolic content in different barley varieties and corresponding malts by liquid chromatography-diode array detection-electrospray ionization tandem mass spectrometry. *Antioxidants*.

[36] Kammerer B., Kahlich R., Biegert C., Gleiter C. H., Heide L. (2005). HPLC-MS/MS analysis of willow bark extracts contained in pharmaceutical preparations. *Phytochemical Analysis*.

[37] Finger D., Machado C. S., Torres Y. R. (2013). Antifungal bioassay-guided fractionation of an oil extract of propolis. *Journal of Food Quality*.

[38] Greenaway W., Whatley F. R. (1990). Analysis of phenolics of bud exudate of *Populus angustifolia* by GC-MS. *Phytochemistry*.

[39] Fernandez M. P., Watson P. A., Breuil C. (2001). Gas chromatography-mass spectrometry method for the simultaneous determination of wood extractive compounds in quaking aspen. *Journal of Chromatography A*.

[40] Rubert-Nason K., Keefover-Ring K., Lindroth R. L. (2018). Purification and analysis of salicinoids. *Current Analytical Chemistry*.

[41] Sabaa M., Elfayoumi H. M., Elshazly S., Youns M., Barakat W. (2017). Anticancer activity of salicin and fenofibrate. *Naunyn-Schmiedeberg’s Archives of Pharmacology*.

[42] Ishikawa T., Nishigaya K., Takami K., Uchikoshi H., Chen I. S., Tsai I. L. (2004). Isolation of salicin derivatives from *Homalium cochinchinensis* and their antiviral activities. *Journal of Natural Products*.

[43] Wang T. Y., Li Q., Bi K. S. (2018). Bioactive flavonoids in medicinal plants: structure, activity and biological fate. *Asian Journal of Pharmaceutical Sciences*.

[44] Debbache N., Atmani D., Atmani D. (2014). Chemical analysis and biological activities of *Populus nigra*, flower buds extracts as source of propolis in Algeria. *Industrial Crops and Products*.

[45] Li Y. X., Zhang W., Sun N. N., Wang X., Feng Y. Q., Zhang X. Y. (2020). Identification and functional verification of differences in phenolic compounds between resistant and susceptible *populus* species. *Phytopathology*.

[46] Li Y., Wang G., Wei T., Fan Z., Yan P. (2016). Nitrogen and sulfur co-doped porous carbon nanosheets derived from willow catkin for supercapacitors. *Nano Energy*.

[47] Wu Y., Wu X., Shi T. (2019). The microstructure and mechanical properties of poplar catkin fibers evaluated by atomic force microscope (AFM) and Nanoindentation. *Forests*.

[48] Darwish R. S., Shawky E., Nassar K. M. (2021). Differential anti-inflammatory biomarkers of the desert truffles *Terfezia claveryi* and *Tirmania nive*a revealed via UPLC-QqQ-MS-based metabolomics combined to chemometrics. *LWT-Food Science and Technology*.

[49] Cheng G. F., Liu D. P., Yang D. X., He K. Q., Bai J. Y., Zhu X. Y. (1994). Antiinflammatory effects of tremulacin, a salicin-related substance isolated from *Populus tomentosa* Carr. leaves. *Phytomedicine*.

