# Supplementary material for: Gender Discrimination of Flower Buds of Mature Populus tomentosa by HPLC Fingerprint Combined with Chemometrics
Source: Int J Anal Chem. 2022 Sep 29;2022:1281521. doi: 10.1155/2022/1281521 (PMC9536970; doi:10.1155/2022/1281521)
Supplement: Supplementary Materials — Table S1: similarity evaluation of 11 male flower buds (MFB) of P. tomentosa. Table S2: similarity evaluation of 11 female flower buds (FFB) of P. tomentosa. Table S3: similarity evaluation of 22 flower buds of P. tomentosa. Table S4: other compounds of the sample F4 identified by UPLC-Q-TOF/MS. Figure S1: optimization of the sample preparation: (A) optimization of the extraction solvents; (B) optimization of the sample-to-solvent ratios; (C) optimization of the extraction methods; the picture above depicts reflux extraction, while the picture below depicts ultrasonic extraction; and (D) optimization of the extraction time. Figure S2: optimization of HPLC Conditions: (A) optimization of the mobile phases: the picture on the left depicts the methanol-water of the mobile phase, and the picture on the right depicts the methanol-0.1% formic acid in the water of the mobile phase; (B) optimization of the analytical columns: the B1 is about the column of Diamonsil C18 (1), the B2 is about the column of Diamonsil C18 (2), the B3 is about the column of Diamonsil Plus C18, the B4 is about the column of Agilent 5 TC-C18 (2), and the B5 is about the column of Agilent ZORBAX SB-C18. Figure S3: the MS1 and MS2 spectrograms of the sample in the positive and negative mode: (A) the MS1 spectrogram at 3.56 min in the positive mode; (B) the MS2 spectrogram at 3.56 min in the positive mode; the source ion of MS2 is 309.0961 m/z; (C) the MS1 spectrogram in the negative mode; and (D) the MS2 spectrogram in the negative mode. Figure S4: the MS1 and MS2 spectrograms of the sample in the positive and negative mode: (A) the MS1 spectrogram at 4.01 min in the positive mode; (B) the MS2 spectrogram at 4.01 min in the positive mode; the source ion of MS2 is 291.0891 m/z; (C) the MS1 spectrogram in the negative mode; and (D) the MS2 spectrogram in the negative mode. Figure S5: the MS1 and MS2 spectrograms of the sample in the positive and negative mode: (A) the MS1 spectrogram at 7.55 min in the [file 1281521.f1.docx]

**Supplementary Materials**

**Gender discrimination of flower buds of mature *Populus tomentosa* by HPLC fingerprint combined with chemometrics**

Zhuojun Li ^1^, Cui Wu ^1^, Bo Xu ^1^, Huijun Wang ^1^, Pingping Song ^1^, Zhenying Liu ^1^, and Zhimao Chao ^1, *^

1 Institute of Chinese Materia Medica, China Academy of Chinese Medical Sciences, Beijing 100700, China

* Correspondence should be addressed to Zhimao Chao; chaozhimao@163.com

Table S1. Similarity evaluation of 11 male flower buds (MFB) of *P. tomentosa*.

|  | M1 | M2 | M3 | M4 | M5 | M6 | M7 | M8 | M9 | M10 | M11 | R_m_ |
| --- | --- | --- | --- | --- | --- | --- | --- | --- | --- | --- | --- | --- |
| M1 | 1.000 | 0.916 | 0.878 | 0.970 | 0.921 | 0.938 | 0.914 | 0.953 | 0.949 | 0.972 | 0.955 | 0.971 |
| M2 | 0.916 | 1.000 | 0.986 | 0.976 | 0.989 | 0.960 | 0.966 | 0.892 | 0.880 | 0.931 | 0.918 | 0.968 |
| M3 | 0.878 | 0.986 | 1.000 | 0.952 | 0.981 | 0.939 | 0.945 | 0.857 | 0.848 | 0.898 | 0.883 | 0.943 |
| M4 | 0.970 | 0.976 | 0.952 | 1.000 | 0.966 | 0.951 | 0.940 | 0.929 | 0.917 | 0.962 | 0.937 | 0.979 |
| M5 | 0.921 | 0.989 | 0.981 | 0.966 | 1.000 | 0.981 | 0.983 | 0.896 | 0.896 | 0.929 | 0.936 | 0.975 |
| M6 | 0.938 | 0.960 | 0.939 | 0.951 | 0.981 | 1.000 | 0.992 | 0.915 | 0.927 | 0.945 | 0.966 | 0.982 |
| M7 | 0.914 | 0.966 | 0.945 | 0.940 | 0.983 | 0.992 | 1.000 | 0.897 | 0.905 | 0.927 | 0.955 | 0.972 |
| M8 | 0.953 | 0.892 | 0.857 | 0.929 | 0.896 | 0.915 | 0.897 | 1.000 | 0.995 | 0.981 | 0.977 | 0.968 |
| M9 | 0.949 | 0.880 | 0.848 | 0.917 | 0.896 | 0.927 | 0.905 | 0.995 | 1.000 | 0.976 | 0.987 | 0.967 |
| M10 | 0.972 | 0.931 | 0.898 | 0.962 | 0.929 | 0.945 | 0.927 | 0.981 | 0.976 | 1.000 | 0.978 | 0.984 |
| M11 | 0.955 | 0.918 | 0.883 | 0.937 | 0.936 | 0.966 | 0.955 | 0.977 | 0.987 | 0.978 | 1.000 | 0.985 |
| R_m_ | 0.971 | 0.968 | 0.943 | 0.979 | 0.975 | 0.982 | 0.972 | 0.968 | 0.967 | 0.984 | 0.985 | 1.000 |

Rm refers to the control fingerprint of male samples.

Table S2. Similarity evaluation of 11 female flower buds (FFB) of *P. tomentosa*.

|  | F1 | F2 | F3 | F4 | F5 | F6 | F7 | F8 | F9 | F10 | F11 | R_f_ |
| --- | --- | --- | --- | --- | --- | --- | --- | --- | --- | --- | --- | --- |
| F1 | 1.000 | 0.967 | 0.992 | 0.968 | 0.993 | 0.971 | 0.993 | 0.969 | 0.987 | 0.983 | 0.989 | 0.990 |
| F2 | 0.967 | 1.000 | 0.988 | 0.996 | 0.966 | 0.986 | 0.947 | 0.993 | 0.987 | 0.988 | 0.990 | 0.991 |
| F3 | 0.992 | 0.988 | 1.000 | 0.988 | 0.990 | 0.988 | 0.981 | 0.984 | 0.996 | 0.995 | 0.996 | 0.999 |
| F4 | 0.968 | 0.996 | 0.988 | 1.000 | 0.964 | 0.995 | 0.953 | 0.997 | 0.985 | 0.985 | 0.989 | 0.992 |
| F5 | 0.993 | 0.966 | 0.990 | 0.964 | 1.000 | 0.963 | 0.980 | 0.960 | 0.993 | 0.991 | 0.991 | 0.988 |
| F6 | 0.971 | 0.986 | 0.988 | 0.995 | 0.963 | 1.000 | 0.965 | 0.993 | 0.981 | 0.979 | 0.982 | 0.991 |
| F7 | 0.993 | 0.947 | 0.981 | 0.953 | 0.980 | 0.965 | 1.000 | 0.958 | 0.970 | 0.964 | 0.972 | 0.978 |
| F8 | 0.969 | 0.993 | 0.984 | 0.997 | 0.960 | 0.993 | 0.958 | 1.000 | 0.978 | 0.979 | 0.985 | 0.990 |
| F9 | 0.987 | 0.987 | 0.996 | 0.985 | 0.993 | 0.981 | 0.970 | 0.978 | 1.000 | 0.999 | 0.997 | 0.996 |
| F10 | 0.983 | 0.988 | 0.995 | 0.985 | 0.991 | 0.979 | 0.964 | 0.979 | 0.999 | 1.000 | 0.998 | 0.996 |
| F11 | 0.989 | 0.990 | 0.996 | 0.989 | 0.991 | 0.982 | 0.972 | 0.985 | 0.997 | 0.998 | 1.000 | 0.998 |
| R_f_ | 0.990 | 0.991 | 0.999 | 0.992 | 0.988 | 0.991 | 0.978 | 0.990 | 0.996 | 0.996 | 0.998 | 1.000 |

R_f_ refers to the control fingerprint of female samples.

Table S3. Similarity evaluation of 22 flower buds of *P. tomentosa*.

|  | F1 | F2 | F3 | F4 | F5 | F6 | F7 | F8 | F9 | F10 | F11 | M1 | M2 | M3 | M4 | M5 | M6 | M7 | M8 | M9 | M10 | M11 | Rn |
| --- | --- | --- | --- | --- | --- | --- | --- | --- | --- | --- | --- | --- | --- | --- | --- | --- | --- | --- | --- | --- | --- | --- | --- |
| F1 | 1.000 | 0.967 | 0.992 | 0.968 | 0.993 | 0.971 | 0.993 | 0.969 | 0.987 | 0.983 | 0.989 | 0.694 | 0.865 | 0.853 | 0.780 | 0.845 | 0.785 | 0.837 | 0.638 | 0.617 | 0.710 | 0.704 | 0.930 |
| F2 | 0.967 | 1.000 | 0.988 | 0.996 | 0.966 | 0.986 | 0.947 | 0.993 | 0.987 | 0.988 | 0.990 | 0.696 | 0.840 | 0.826 | 0.750 | 0.846 | 0.823 | 0.873 | 0.636 | 0.634 | 0.707 | 0.733 | 0.933 |
| F3 | 0.992 | 0.988 | 1.000 | 0.988 | 0.990 | 0.988 | 0.981 | 0.984 | 0.996 | 0.995 | 0.996 | 0.700 | 0.856 | 0.844 | 0.772 | 0.848 | 0.803 | 0.857 | 0.637 | 0.624 | 0.709 | 0.718 | 0.937 |
| F4 | 0.968 | 0.996 | 0.988 | 1.000 | 0.964 | 0.995 | 0.953 | 0.997 | 0.985 | 0.985 | 0.989 | 0.709 | 0.838 | 0.821 | 0.758 | 0.844 | 0.819 | 0.868 | 0.638 | 0.636 | 0.711 | 0.735 | 0.935 |
| S5 | 0.993 | 0.966 | 0.990 | 0.964 | 1.000 | 0.963 | 0.980 | 0.960 | 0.993 | 0.991 | 0.991 | 0.670 | 0.862 | 0.857 | 0.765 | 0.846 | 0.782 | 0.840 | 0.628 | 0.608 | 0.691 | 0.695 | 0.924 |
| F6 | 0.971 | 0.986 | 0.988 | 0.995 | 0.963 | 1.000 | 0.965 | 0.993 | 0.981 | 0.979 | 0.982 | 0.712 | 0.831 | 0.808 | 0.759 | 0.833 | 0.799 | 0.848 | 0.629 | 0.62 | 0.705 | 0.717 | 0.928 |
| F7 | 0.993 | 0.947 | 0.981 | 0.953 | 0.980 | 0.965 | 1.000 | 0.958 | 0.970 | 0.964 | 0.972 | 0.715 | 0.873 | 0.857 | 0.802 | 0.851 | 0.784 | 0.830 | 0.640 | 0.616 | 0.718 | 0.700 | 0.926 |
| F8 | 0.969 | 0.993 | 0.984 | 0.997 | 0.960 | 0.993 | 0.958 | 1.000 | 0.978 | 0.979 | 0.985 | 0.729 | 0.853 | 0.831 | 0.776 | 0.856 | 0.830 | 0.874 | 0.654 | 0.649 | 0.727 | 0.744 | 0.941 |
| F9 | 0.987 | 0.987 | 0.996 | 0.985 | 0.993 | 0.981 | 0.970 | 0.978 | 1.000 | 0.999 | 0.997 | 0.678 | 0.851 | 0.847 | 0.756 | 0.846 | 0.798 | 0.854 | 0.626 | 0.615 | 0.695 | 0.709 | 0.931 |
| F10 | 0.983 | 0.988 | 0.995 | 0.985 | 0.991 | 0.979 | 0.964 | 0.979 | 0.999 | 1.000 | 0.998 | 0.677 | 0.848 | 0.844 | 0.752 | 0.847 | 0.800 | 0.857 | 0.627 | 0.618 | 0.691 | 0.711 | 0.930 |
| F11 | 0.989 | 0.990 | 0.996 | 0.989 | 0.991 | 0.982 | 0.972 | 0.985 | 0.997 | 0.998 | 1.000 | 0.693 | 0.859 | 0.852 | 0.766 | 0.854 | 0.810 | 0.863 | 0.640 | 0.630 | 0.707 | 0.723 | 0.938 |
| M1 | 0.694 | 0.696 | 0.700 | 0.709 | 0.670 | 0.712 | 0.715 | 0.729 | 0.678 | 0.677 | 0.693 | 1.000 | 0.916 | 0.878 | 0.970 | 0.921 | 0.938 | 0.914 | 0.953 | 0.949 | 0.972 | 0.955 | 0.892 |
| M2 | 0.865 | 0.840 | 0.856 | 0.838 | 0.862 | 0.831 | 0.873 | 0.853 | 0.851 | 0.848 | 0.859 | 0.916 | 1.000 | 0.986 | 0.976 | 0.989 | 0.960 | 0.966 | 0.892 | 0.880 | 0.931 | 0.918 | 0.970 |
| M3 | 0.853 | 0.826 | 0.844 | 0.821 | 0.857 | 0.808 | 0.857 | 0.831 | 0.847 | 0.844 | 0.852 | 0.878 | 0.986 | 1.000 | 0.952 | 0.981 | 0.939 | 0.945 | 0.857 | 0.848 | 0.898 | 0.883 | 0.950 |
| M4 | 0.780 | 0.750 | 0.772 | 0.758 | 0.765 | 0.759 | 0.802 | 0.776 | 0.756 | 0.752 | 0.766 | 0.970 | 0.976 | 0.952 | 1.000 | 0.966 | 0.951 | 0.940 | 0.929 | 0.917 | 0.962 | 0.937 | 0.932 |
| M5 | 0.845 | 0.846 | 0.848 | 0.844 | 0.846 | 0.833 | 0.851 | 0.856 | 0.846 | 0.847 | 0.854 | 0.921 | 0.989 | 0.981 | 0.966 | 1.000 | 0.981 | 0.983 | 0.896 | 0.896 | 0.929 | 0.936 | 0.971 |
| M6 | 0.785 | 0.823 | 0.803 | 0.819 | 0.782 | 0.799 | 0.784 | 0.830 | 0.798 | 0.800 | 0.810 | 0.938 | 0.960 | 0.939 | 0.951 | 0.981 | 1.000 | 0.992 | 0.915 | 0.927 | 0.945 | 0.966 | 0.953 |
| M7 | 0.837 | 0.873 | 0.857 | 0.868 | 0.840 | 0.848 | 0.830 | 0.874 | 0.854 | 0.857 | 0.863 | 0.914 | 0.966 | 0.945 | 0.940 | 0.983 | 0.992 | 1.000 | 0.897 | 0.905 | 0.927 | 0.955 | 0.974 |
| M8 | 0.638 | 0.636 | 0.637 | 0.638 | 0.628 | 0.629 | 0.640 | 0.654 | 0.626 | 0.627 | 0.640 | 0.953 | 0.892 | 0.857 | 0.929 | 0.896 | 0.915 | 0.897 | 1.000 | 0.995 | 0.981 | 0.977 | 0.857 |
| M9 | 0.617 | 0.634 | 0.624 | 0.636 | 0.608 | 0.620 | 0.616 | 0.649 | 0.615 | 0.618 | 0.630 | 0.949 | 0.880 | 0.848 | 0.917 | 0.896 | 0.927 | 0.905 | 0.995 | 1.000 | 0.976 | 0.987 | 0.851 |
| M10 | 0.710 | 0.707 | 0.709 | 0.711 | 0.691 | 0.705 | 0.718 | 0.727 | 0.695 | 0.691 | 0.707 | 0.972 | 0.931 | 0.898 | 0.962 | 0.929 | 0.945 | 0.927 | 0.981 | 0.976 | 1.000 | 0.978 | 0.903 |
| M11 | 0.704 | 0.733 | 0.718 | 0.735 | 0.695 | 0.717 | 0.700 | 0.744 | 0.709 | 0.711 | 0.723 | 0.955 | 0.918 | 0.883 | 0.937 | 0.936 | 0.966 | 0.955 | 0.977 | 0.987 | 0.978 | 1.000 | 0.909 |
| Rn | 0.930 | 0.933 | 0.937 | 0.935 | 0.924 | 0.928 | 0.926 | 0.941 | 0.931 | 0.930 | 0.938 | 0.892 | 0.970 | 0.950 | 0.932 | 0.971 | 0.953 | 0.974 | 0.857 | 0.851 | 0.903 | 0.909 | 1.000 |

R_n_ refers to the control fingerprint of samples.

Table S4. Other compounds of the sample F4 identified by UPLC-Q-TOF/MS.

| **Rt (min)** | **Assignment** | **Formula** | **ESI (+)** | | **ESI (-)** | | **Reference** |
| --- | --- | --- | --- | --- | --- | --- | --- |
|  |  |  | **MS^1^ (ppm)** | **MS^2^** | **MS^1^ (ppm)** | **MS^2^** |  |
| 3.56 | Salicin ^b^ | C_13_H_18_O_7_ | 309.0961 [M+Na]^+^ (1.1) | 291.0825; 201.0126 | 285.0973 [M-H]^-^ (-0.4) | 123.0444; 121.0285 | [35] |
| 4.01 | Catechin ^b^ | C_15_H_14_O_6_ | 291.0891 [M+H]^+^ (2.2); 313.0695 [M+Na]^+^ (0.7) | 139.0421 | 289.0712 [M-H]^-^ (0.7) | 245.0811; 205.0495; 179.0330 | [36] |
| 7.55 | Salicortin ^b^ | C_20_H_24_O_10_ | 447.1261 [M+Na]^+^ (-0.6); 463.0991 [M+Ka]^+^ (-1.6); 425.1415 [M+H]^+^ (-3.3) | 309.0943; 179.0356 | 423.1297 [M-H]^-^ (1.4) | 285.0975; 155.0340; 137.0233; 123.0443; 121.0289 | [37] |
| 10.47 | Isograndidentatin A ^b^ | C_21_H_28_O_9_ | 447.1648 [M+Na]^+^ (1.7); 463.1405 [M+Ka]^+^ (3.5); 425.1798 [M+H]^+^ (-1.4) | 309.0967; 147.0476 | 423.1656 [M-H]^-^ (0.2) | 307.0818; 163.0390; 145.0285 | [21] |
| 10.65 | Isograndidentatin B ^b^ | C_21_H_28_O_9_ | 447.1642 [M+Na]^+^ (1.1); 463.1368 [M+Ka]^+^ (-0.2);  425.1831 [M+H]^+^ (1.9) | 309.1007; 147.0471 | 423.1654 [M-H]^-^ (-0.2) | 307.0803; 163.0389; 145.0285 |  |
| 10.92 | Grandidentatin ^b^ | C_21_H_28_O_9_ | 447.1641 [M+Na]^+^ (1.0); 463.1394 [M+Ka]^+^ (2.4); 425.1808 [M+H]^+^ (-0.4) | 309.0995; 147.0476 | 423.1656 [M-H]^-^ (0.2) | 307.0816; 163.0391; 145.0286 |  |
| 11.21 | Tremuloidin ^b^ | C_20_H_22_O_8_ | 413.1221 [M+Na]^+^ (0.9); 429.0968 [M+K]^+^ (1.6) | 289.0710; 160.9956 | 389.1423 [M-H]^-^ (1.8) | 123.0444; 121.0284 | [37] |
| 16.01 | Sakuranetin ^b^ | C_16_H_14_O_5_ | 287.0927 [M+H]^+^ (0.8) | 167.0376; 147.0492 | 285.0766 [M-H]^-^ (1.1) | 165.0184; 119.0495; 93.0340 | [38] |
| 16.07 | Isosakuranetin ^b^ | C_16_H_14_O_5_ | 287.0934 [M+H]^+^ (1.5) | 153.0214 | 285.0767 [M-H]^-^ (1.4) | 243.0658; 164.0105; 151.0025; 136.0158 | [39] |
| 18.71 | Benzyl Caffeate ^b^ | C_16_H_14_O_4_ | 271.0992 [M+H]^+^ (2.2) | - | - | - | [38] |
| 19.81 | 3-Phenylpropanoic acid ^b^ | C_9_H_10_O_2_ | 301.1429 [2M+H]^+^ (-1.1) | - | - | - | [40] |
| 20.00 | 4-Hydroxy-2-methylacetophenone ^b^ | C_9_H_10_O_2_ | 301.1441 [2M+H]^+^ (0.1) | - | - | - | [41] |

b Identified by comparing with literature


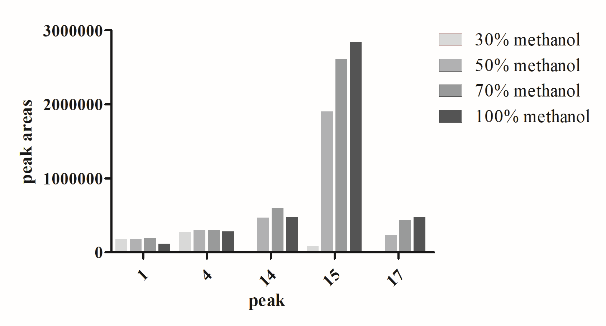

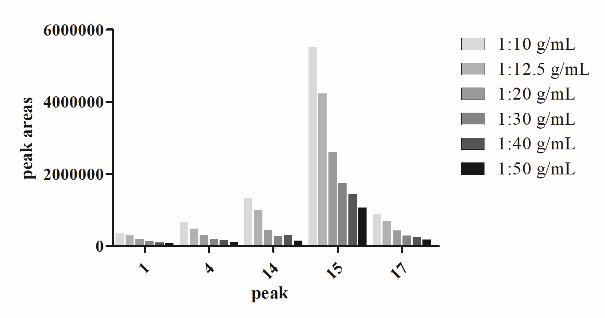


(A) (B)


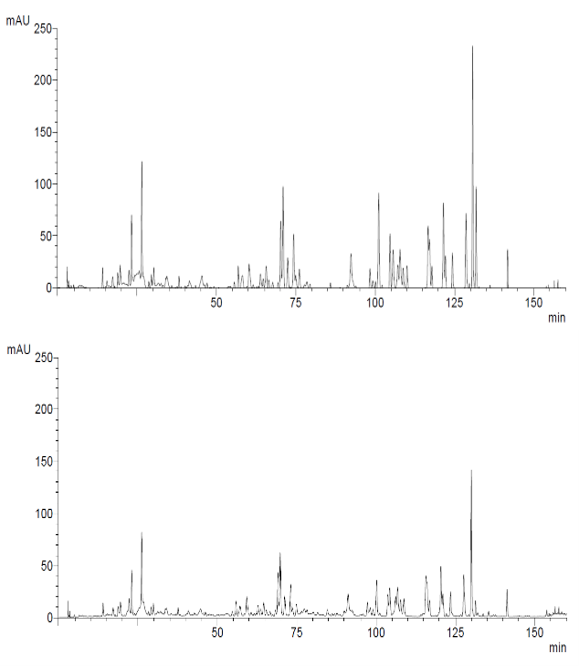

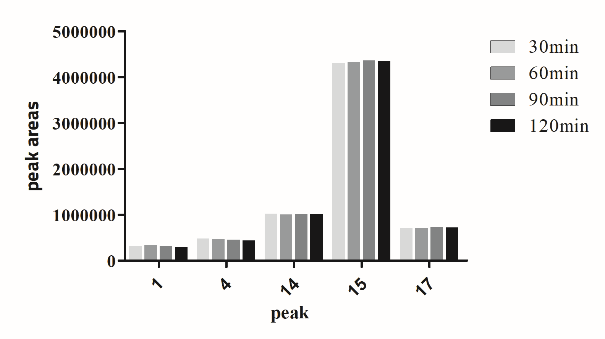


(C) (D)

Figure S1 Optimization of sample preparation. (A) optimization of the extraction solvents; (B) optimization of the ratios of sample to solvent; (C) optimization of the extraction methods; the picture above is about the reflux extraction; and the picture below is about the ultrasonic extraction; (D) optimization of the extraction time.


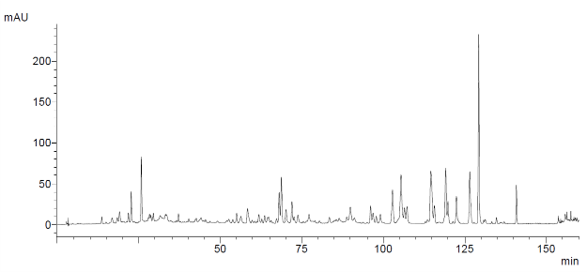

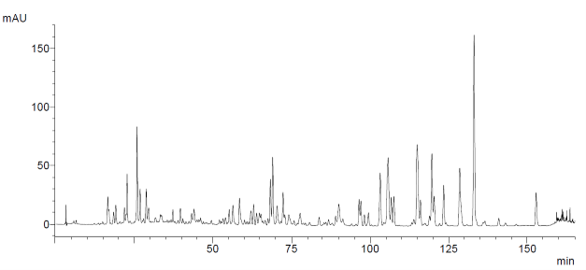


(A)


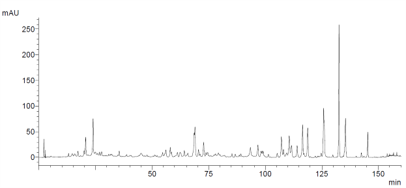

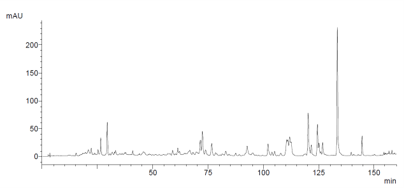


(B1) (B2)


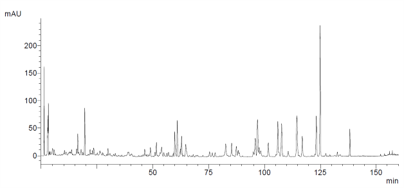

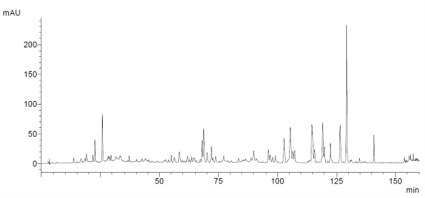


(B3) (B4)


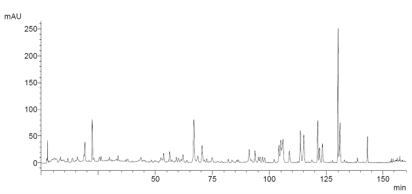


(B5)

Figure S2 Optimization of HPLC Conditions. (A) optimization of the mobile phases; the picture on the left is about the methanol-water of mobile phase; and the picture on the right is about the methanol-0.1% formic acid in water of mobile phase; (B) optimization of the analytical columns; the B1 is about the column of Diamonsil C_18_(1); the B2 is about the column of Diamonsil C_18_(2); the B3 is about the column of Diamonsil Plus C_18_; the B4 is about the column of Agilent 5 TC-C_18_(2); the B5 is about the column of Agilent ZORBAX SB-C_18_.

(B)

(A)

(D)

(C)

Figure S3. The MS^1^ and MS^2^ spectrograms of sample in the positive and negative mode. (A) the MS^1^ spectrogram at 3.56 min in the positive mode; (B) the MS^2^ spectrogram at 3.56 min in the positive mode; the source ion of MS^2^ is 309.0961 *m/z*; (C) the MS^1^ spectrogram in the negative mode; (D) the MS^2^ spectrogram in the negative mode.

(B)

(A)

(D)

(C)

Figure S4. The MS^1^ and MS^2^ spectrograms of sample in the positive and negative mode. (A) the MS^1^ spectrogram at 4.01 min in the positive mode; (B) the MS^2^ spectrogram at 4.01 min in the positive mode; the source ion of MS^2^ is 291.0891 *m/z*; (C) the MS^1^ spectrogram in the negative mode; (D) the MS^2^ spectrogram in the negative mode.

(B)

(A)

(D)

(C)

Figure S5. The MS^1^ and MS^2^ spectrograms of sample in the positive and negative mode. (A) the MS^1^ spectrogram at 7.55 min in the positive mode; (B) the MS^2^ spectrogram at 7.55 min in the positive mode; the source ion of MS^2^ is 425.1415 *m/z*; (C) the MS^1^ spectrogram in the negative mode; (D) the MS^2^ spectrogram in the negative mode.

(B)

(A)

(D)

(C)

(F)

(E)





(G)

Figure S6. The MS^1^ and MS^2^ spectrograms of siebolside B and sample in the positive and negative mode. (A) The MS^1^ spectrogram of siebolside B in the positive mode; (B) the MS^2^ spectrogram of siebolside B in the positive mode; the source ion of MS^2^ is 407.1359 *m/z*; (C) the MS^1^ spectrogram of peak No.4 at 8.91 min in sample in the positive mode; (D) the MS^2^ spectrogram of peak No.4 at 8.91 min in sample in the positive mode; the source ion of MS^2^ is 407.1313 *m/z*; (E) the MS^1^ spectrogram in sample in the negative mode; (F) the MS^2^ spectrogram in sample in the negative mode; (G) the structure of siebolside B.

(B)

(A)

(D)

(C)

Figure S7. The MS and MS/MS spectrograms of sample in the positive and negative mode. (A) the MS spectrogram at 10.47 min in the positive mode; (B) the MS/MS spectrogram at 10.47 min in the positive mode; the source ion of MS^2^ is 425.1798 *m/z*; (C) the MS^1^ spectrogram in the negative mode; (D) the MS^2^ spectrogram in the negative mode.

(B)

(A)

(D)

(C)

Figure S8. The MS^1^ and MS^2^ spectrograms of sample in the positive and negative mode. (A) the MS^1^ spectrogram at 10.65 min in the positive mode; (B) the MS^2^ spectrogram at 10.65 min in the positive mode; the source ion of MS^2^ is 425.1831 *m/z*; (C) the MS^1^ spectrogram in the negative mode; (D) the MS^2^ spectrogram in the negative mode.

(B)

(A)

(D)

(C)

Figure S9. The MS^1^ and MS^2^ spectrograms of sample in the positive and negative mode. (A) the MS^1^ spectrogram at 10.92 min in the positive mode; (B) the MS^2^ spectrogram at 10.92 min in the positive mode; the source ion of MS^2^ is 425.1808 *m/z*; (C) the MS^1^ spectrogram in the negative mode; (D) the MS^2^ spectrogram in the negative mode.

(B)

(A)

(D)

(C)

Figure S10. The MS^1^ and MS^2^ spectrograms of sample in the positive and negative mode. (A) the MS^1^ spectrogram at 11.21 min in the positive mode; (B) the MS^2^ spectrogram at 11.21 min in the positive mode; the source ion of MS^2^ is 413.1221 *m/z*; (C) the MS^1^ spectrogram in the negative mode; (D) the MS^2^ spectrogram in the negative mode.

(B)

(A)

(D)

(C)





(E)

Figure S11. The MS^1^ and MS^2^ spectrograms of sample in the positive and negative mode. (A) the MS^1^ spectrogram of peak 9 at 13.76 min in the positive mode; (B) the MS^2^ spectrogram of peak 9 at 13.76 min in the positive mode; the source ion of MS^2^ is 551.1531 *m/z*; (C) the MS^1^ spectrogram in the negative mode; (D) the MS^2^ spectrogram in the negative mode; (E) the structure of peak 9.

(B)

(A)

(D)

(C)

Figure S12. The MS^1^ and MS^2^ spectrograms of sample in the positive and negative mode. (A) the MS^1^ spectrogram at 16.01 min in the positive mode; (B) the MS^2^ spectrogram at 16.01 min in the positive mode; the source ion of MS^2^ is 287.0927 *m/z*; (C) the MS^1^ spectrogram in the negative mode; (D) the MS^2^ spectrogram in the negative mode.

(B)

(A)

(D)

(C)

Figure S13. The MS^1^ and MS^2^ spectrograms of sample in the positive and negative mode. (A) the MS^1^ spectrogram at 16.07 min in the positive mode; (B) the MS^2^ spectrogram at 16.07 min in the positive mode; the source ion of MS^2^ is 287.0934 *m/z*; (C) the MS^1^ spectrogram in the negative mode; (D) the MS^2^ spectrogram in the negative mode.

(B)

(A)

Figure S14. The MS^1^ and MS^2^ spectrograms of sample in the positive mode. (A) the MS^1^ spectrogram of peak 14 at 17.37 min; (B) the MS^2^ spectrogram of peak 14 at 17.37 min the source ion of MS^2^ is 496.1318 *m/z*.

(B)

(A)

Figure S15. The MS^1^ and MS^2^ spectrograms of sample in the positive mode. (A) the MS^1^ spectrogram of peak 15 at 18.14 min; (B) the MS^2^ spectrogram of peak 15 at 18.14 min; the source ion of MS^2^ is 318.3023 *m/z*.

(B)

(A)

Figure S16. The MS^1^ and MS^2^ spectrograms of sample in the positive mode. (A) the MS^1^ spectrograms at 18.71 min; (B) the MS^2^ spectrogram at 18.71 min.

(B)

(A)

Figure S17. The MS^1^ and MS^2^ spectrograms of sample in the positive mode. (A) the MS^1^ spectrograms at 19.81 min; (B) the MS^2^ spectrogram at 19.81 min.

(B)

(A)

Figure S18. The MS^1^ and MS^2^ spectrograms of sample in the positive mode. (A) the MS^1^ spectrograms at 20.00 min; (B) the MS^2^ spectrogram at 20.00 min.
